# Probiotic and technological properties of *Lactobacillus* spp. strains from the human stomach in the search for potential candidates against gastric microbial dysbiosis

**DOI:** 10.3389/fmicb.2014.00766

**Published:** 2015-01-14

**Authors:** Susana Delgado, Analy M. O. Leite, Patricia Ruas-Madiedo, Baltasar Mayo

**Affiliations:** Departamento de Microbiología y Bioquímica, Instituto de Productos Lácteos de Asturias (IPLA), Consejo Superior de Investigaciones Científicas (CSIC)Villaviciosa, Spain

**Keywords:** stomach microbiota, gastric lactobacilli, specific probiotics, functional characterization, antioxidative activity, anti-*Helicobacter* activity, fermentation capability

## Abstract

This work characterizes a set of lactobacilli strains isolated from the stomach of healthy humans that might serve as probiotic cultures. Ten different strains were recognized by rep-PCR and PFGE fingerprinting among 19 isolates from gastric biopsies and stomach juice samples. These strains belonged to five species, *Lactobacillus gasseri* (3), *Lactobacillus reuteri* (2), *Lactobacillus vaginalis* (2)*, Lactobacillus fermentum* (2) and *Lactobacillus casei* (1). All ten strains were subjected to a series of *in vitro* tests to assess their functional and technological properties, including acid resistance, bile tolerance, adhesion to epithelial gastric cells, production of antimicrobial compounds, inhibition of *Helicobacter pylori*, antioxidative activity, antibiotic resistance, carbohydrate fermentation, glycosidic activities, and ability to grow in milk. As expected, given their origin, all strains showed good resistance to low pH (3.0), with small reductions in counts after 90 min exposition to this pH. Species- and strain-specific differences were detected in terms of the production of antimicrobials, antagonistic effects toward *H. pylori*, antioxidative activity and adhesion to gastric epithelial cells. None of the strains showed atypical resistance to a series of 16 antibiotics of clinical and veterinary importance. Two *L. reuteri* strains were deemed as the most appropriate candidates to be used as potential probiotics against microbial gastric disorders; these showed good survival under gastrointestinal conditions reproduced *in vitro*, along with strong anti-*Helicobacter* and antioxidative activities. The two *L. reuteri* strains further displayed appropriated technological traits for their inclusion as adjunct functional cultures in fermented dairy products.

## Introduction

Over recent decades, culture-independent techniques have revealed the stomach to be home to a well-adapted, niche-specific microbial community (Bik et al., 2006; Andersson et al., 2008; Delgado et al., 2013). Gastric microbial communities are of potential probiotic use in the treatment of several diseases, but only a few studies have attempted to cultivate their members (Adamson et al., 1999; Li et al., 2009; Delgado et al., 2013). The isolation and characterization of stomach originated strains could provide novel probiotic candidates with enhanced capacities to counteract gastric pathogens such as *Helicobacter pylori* (Cui et al., 2010). This Gram-negative, microaerophilic microorganism infects over 50% of the population worldwide (Bruce and Maaroos, 2008). Indeed, *H. pylori* is the most important aetiological agent in chronic gastritis, peptic ulcers and gastric cancer (Peek and Blaser, 2002). The eradication (efficiency 80–90%) of *H. pylori* is possible using a combination of antibiotics and antacids. However, side-effects are common. As an alternative or complementary therapy, or indeed as a preventive strategy, use might be made of probiotics for the management of this infection (Malfertheiner et al., 2007). To date, studies have focused on conventional probiotic cultures from different origins. Among these, *Lactobacillus reuteri* having anti *H. pylori* action is one promising approach (Francavilla et al., 2008).

The aim of the present work was to examine *in vitro* the functional and technological characteristics of *Lactobacillus* isolates recovered from the stomach mucosa and gastric juice of healthy individuals in a previous study (Delgado et al., 2013) for their evaluation as probiotics in functional products against *H. pylori* infection. Gastric candidate probiotic strains with potential application in the prevention and treatment of gastric disorders and dysbiosis were then selected.

## Materials and methods

### Bacterial isolates and growth conditions

Nineteen *Lactobacillus* isolates belonging to five different species were cultured from gastric biopsies and stomach juice samples provided by healthy subjects (see Delgado et al., 2013). Unless otherwise indicated, all isolates were cultured in de Man, Rogosa and Sharpe (MRS; Merck, Darmstadt, Germany) medium supplemented with 0.25% cysteine (Merck) (MRSC). Incubations proceeded at 37°C for 24 h in an anaerobic chamber (Mac500, Down Whitley Scientific, West Yorkshire, UK) containing an anoxic atmosphere (10% H_2_, 10% CO_2_, 80% N_2_).

### Typing of lactobacilli

The isolates were genotyped by repetitive extragenic palindromic (rep)-PCR and by pulsed-field gel electrophoresis (PFGE) fingerprinting. For rep-PCR, total DNA from the isolates was purified from overnight cultures using the GenElute™ Bacterial Genomic DNA Kit (Sigma-Aldrich, St. Louis, MO, USA), following the manufacturer's recommendations. Amplifications were performed using the primer BoxA2-R (5′-ACGTGGTTTGAAGAGATTTTCG-3′), as reported by Koeuth et al. (1995). For PFGE, genomic DNA was isolated and digested at 30°C for 4 h in agarose plugs containing 20 U of the restriction endonuclease SmaI (Boehringer Mannheim, Mannheim, Germany). Electrophoresis was performed in 1% FastLane agarose gels (FMC, Philadelphia, PA, USA) in 0.5X TBE (Tris-borate-EDTA) for 20 h at 14°C and at 6 V/cm, using a CHEF-DRII apparatus (Bio-Rad, Richmond, CA, USA). Pulse times ranged from 0.1 to 2 s for 4 h, from 2 to 5 s for 12 h, and from 5 to 10 s for 4 h. Low-range and bacteriophage lambda ladder PFGE markers (both from New England BioLabs, Ipswich, MA, USA) were included in the gels.

### Gastrointestinal tolerance

#### Viability at low pH

The ability of the isolates to survive under acidic conditions was assessed by exposing the cells to an acidic solution. HCl was added to cell suspensions (≈10^8^ cells/ml) in a sterile saline solution (0.9%) to achieve pHs ranging from 2.0 to 6.5 (pH 7.0 was used as a control). The cells were then incubated at 37°C for 90 min. After incubation the pH of the medium was recorded again and the viability of the strains assessed by plate counting on MRSC.

#### Resistance to bile

The tolerance of the strains to bovine bile (Ox-gall, Sigma-Aldrich) was assayed following the procedure of Charteris et al. (1998). Briefly, individual colonies from MRSC plates were suspended in 2 ml of sterile saline solution until a density corresponding to McFarland standard 1 was obtained. Aliquots of this suspension (10μl) were spotted onto bile-containing agar plates. The concentration of bile assayed ranged from 0.25 to 4% (w/v).

### Production of antimicrobial compounds

#### Anti-Helicobacter pylori activity

*H. pylori* DSM 10242 was routinely grown in solid or liquid brain heart infusion medium (BHI; Oxoid, Basingstoke, Hampshire, UK), supplemented with either 5% (w/v) horse blood (Oxoid) or 10% (w/v) fetal bovine serum (Oxoid), respectively. Inoculated cultures were then incubated at 37°C for 3–7 days under the microaerophilic conditions generated by the CampyGen system (Oxoid). Antagonistic activity of lactobacilli against *H. pylori* was assessed by a broth inhibition assay after growing of the test strains in Elliker broth (Scharlau, Barcelona, Spain), since MRS medium inhibits the growth of this pathogen (Ryan et al., 2008b). Inhibition assays were performed in 96-well, “U”-bottom polystyrene microtitre plates. Supplemented BHI was inoculated at 1.5 (v/v) with a concentrated *H. pylori* culture (OD_600 nm_ 1.0), and the cell suspension was dispensed into the wells of a microtitre plate. Then, 45 μl of the supernatant of each individual lactobacilli strain were added to the wells. As a negative control, aliquots of non-inoculated Elliker medium were also tested. The multi-well plates were then incubated for 3 days under the same conditions as above, and the OD_600 nm_ recorded using a microplate spectrophotometer (Benchmark; Bio-Rad Laboratories, Hercules, CA, USA). Assays were also performed with pH-neutralized supernatants (pH 6.6). All experiments were repeated twice using independent cultures; all supernatants were further assayed in triplicate.

#### Bacteriocins

Bacteriocin production was consecutively examined by an agar spot test and a well-diffusion assay as previously described (Delgado et al., 2007), using two well-recognized bacteriocin-susceptible strains (*Lactobacillus sakei* CECT 906 and *Lactococcus lactis* subsp. *lactis* IL1403) as indicators.

#### Hydrogen peroxide (H_2_O_2_)

H_2_O_2_ production was tested following the procedure described by Song et al. (1999). MRSC agar plates supplemented with 0.25 mg/ml of tetramethylbenzidine (TMB, Sigma-Aldrich) and 0.01 mg/ml of horseradish peroxidase (HRP, Sigma-Aldrich) were inoculated with the strains and incubated at 37°C under both aerobic and anaerobic conditions. The presence of any blue pigment in the H_2_O_2_–producing colonies was recorded after 2 days. *Lactobacillus jensenii* CECT 4306 (Martín and Suárez, 2010) was used as positive control.

#### Reuterin

The presence of the gene coding for the large subunit of glycerol dehydratase, which is essential in the production of reuterin (3-hydroxypropionaldehyde) (Claisse and Lonvaud-Funel, 2001), was checked for in the *Lactobacillus reuteri* strains via PCR. *L. reuteri* CECT 925, a reuterin-producing strain (Martín et al., 2005), was used as positive control.

### Adhesion to an epithelial gastric cell line

The adhesion of the strains to the gastric mucosa was assessed *in vitro* using the gastric cell line AGS (ECACC number 89090402, Sigma-Aldrich), which is derived from a human gastric adenocarcinoma. The latter cells were cultured as per routine in Ham's F12 medium (LabClinics, Barcelona, Spain) supplemented with 2 mM of L-glutamine (PAA Laboratories GmbH, Paschina, Austria), 10% fetal bovine serum, plus 50 μg/ml of penicillin, 50 μg/ml of streptomycin, 50 μg/ml of gentamicin, and 1.25 μg/ml amphotericin B (Sigma-Aldrich). Monolayers of the cells were prepared in 24-well tissue culture plates (Becton Dickinson, New Jersey, USA) at a concentration of 10^5^ cells/ml. After reaching confluence and differentiating (3 + 1 days), the strains were added at a ratio 1:1. For this, bacterial cultures were harvested by centrifugation, washed with Dulbecco's phosphate-buffered saline solution (Sigma-Aldrich), and suspended in Ham's F12 medium without antibiotics. After 1 h of co-incubation at 37°C in a 5% CO_2_ atmosphere, the monolayers were washed 3 times with a phosphate-buffered saline solution to remove any non-attached bacteria. The monolayers were then disrupted with a 0.25% trypsin-EDTA solution (Sigma-Aldrich), and the attached bacteria enumerated by standard dilution and plate counting on MRSC. Experiments were carried out in triplicate and each strain tested in duplicate. The adhesion capacity of the strains was compared to that of the recognized probiotic strain *Lactobacillus rhamnosus* ATCC 53103 (strain GG). The results were expressed as the percentage of adhered bacteria with respect to the initial number of bacteria added.

### Antioxidative activity

The total antioxidative activity (TAA) of the gastric lactobacilli was assessed using the linolenic acid (LA) test, which evaluates the ability to inhibit lipid peroxidation. Bacterial cultures (24 h) were centrifuged, washed twice in saline solution, and suspended in the same solution to an OD_600 nm_ of 1.0. Intact cells in the saline solution were examined, as were lysates obtained using a cell disruptor (Constant Systems, Daventry, UK). Reactions were performed according to the procedure described by Kullisaar et al. (2002), using 45μl samples (whole cells or lysate). The absorbance at 534 nm was measured using a UV-Vis Spectrophotometer (Hitachi High-Technologies, Tokyo, Japan). Intact cells and cell lysates were assayed in triplicate. The results were expressed as the percentage of LA oxidation inhibition.

### Antibiotic resistance

The resistance/susceptibility profiles of the different strains to 16 antibiotics were determined by microdilution using VetMIC™ plates for lactic acid bacteria (LAB) (National Veterinary Institute of Sweden, Uppsala, Sweden). The strains were grown in LSM (Klare et al., 2005) agar plates and then suspended in 2 ml of sterile saline solution to obtain a density corresponding to McFarland standard 1. The suspension was further diluted 1:1000 with LSM, and 100μl of this inoculum were added to each well. The minimum inhibitory concentration (MIC) was defined as the lowest antibiotic concentration at which no visual growth was observed after incubation at 37°C for 48 h.

### Technological traits

#### Fermentation capabilities

The carbohydrate fermentation profile of the strains was initially determined using a miniaturized commercial system (Phene-Plate, Stockholm, Sweden), following the manufacturer's instructions. Growth in the presence of different substrates (lactose, maltose, trehalose, melibiose and raffinose [all from Sigma-Aldrich]) was further evaluated by recording the OD_600 nm_ at 24 and 48 h. The strains were inoculated (1% v/v) into a basal fermentation medium (MRS without glucose) supplemented with 2% (w/v) of the carbohydrate under test. All strains had been previously adapted overnight in the corresponding fermentation broth.

#### Glycosidic activity

Enzyme activities were initially measured using the semi-quantitative API-ZYM system (bioMérieux, Marcy l'Etoile, France), following the manufacturer's instructions. Glycosidic activities in cell-free extracts were confirmed and quantified by enzyme assays using the *p*-nitrophenyl (*p*-NP) derivatives 4-NP-β-D-glucopyranoside, 4-NP-α-D-glucopyranoside, 4-NP-β-D-galactopyranoside and 4-NP-α-D-galactopyranoside (all from Sigma-Aldrich) as substrates. Cells from 20 ml cultures in basal fermentation medium with different inducing carbohydrates were harvested by centrifugation, washed with 100 mM potassium phosphate buffer pH 6.8, and the pellets suspended in 2 ml of the same buffer. Cells were disrupted as above and centrifuged to remove cell debris. The extracts were then assayed for glycosidic activity. Reactions were performed using 800μl of 40 mM buffer acetate pH 5.5, 100μl of the different *p*-NP derivatives at 10 mM, and 100μl of the cell-free extracts. Incubation proceeded for 30 min at 37°C and the reactions were then stopped by adding 1 ml of cold 1 M sodium carbonate. After centrifugation, the absorbance at 410 nm was recorded. The protein content of cell-free extracts was determined using the BCA protein assay kit (Pierce, Rockford, IL, USA). Activities were expressed as specific activity in mU per mg of protein. One unit of activity (1 U) was defined as the amount of protein that released 1 μmol of *p*-NP per min.

#### Growth and acidification of milk

Overnight cultures, previously washed in a sterile saline solution, were used to inoculate UHT milk (CAPSA, Siero, Spain) to provide an initial cell concentration of about 10^7^ cfu/ml. After incubation at 37°C in a 5% CO_2_ atmosphere for 24 and 48 h, bacterial counts were performed on MRSC agar plates. The pH of the inoculated and control milk samples was measured using a Crison pH-meter (Crison Instruments, Barcelona, Spain) and via titration with phenolphthalein according to FIL/IDF Standard 86.

## Results

### Typing of gastric lactobacilli strains

The 19 gastric lactobacilli isolates (9 *Lactobacillus gasseri*, 4 *Lactobacillus reuteri*, 3 *Lactobacillus vaginalis*, 2 *Lactobacillus fermentum* and 1 *Lactobacillus casei*) were successively typed by rep-PCR and PFGE (Supplementary Figure 1). The results of these two techniques agreed well, and 10 different strains were considered based on their distinct electrophoretic profiles provided by both methods. The ten selected strains included three *L. gasseri* strains (LG52, LG102, and LG123) from two subjects, two *L. reuteri* strains (LR32 and LR34) from a single subject, two *L. vaginalis* strains (LV51 and LV121) from different subjects, two genetically unrelated *L. fermentum* strains (LF1 and LF2) from a single subject, and a single strain of *L. casei* (LC71).

### Functional properties

**Figure 1:**
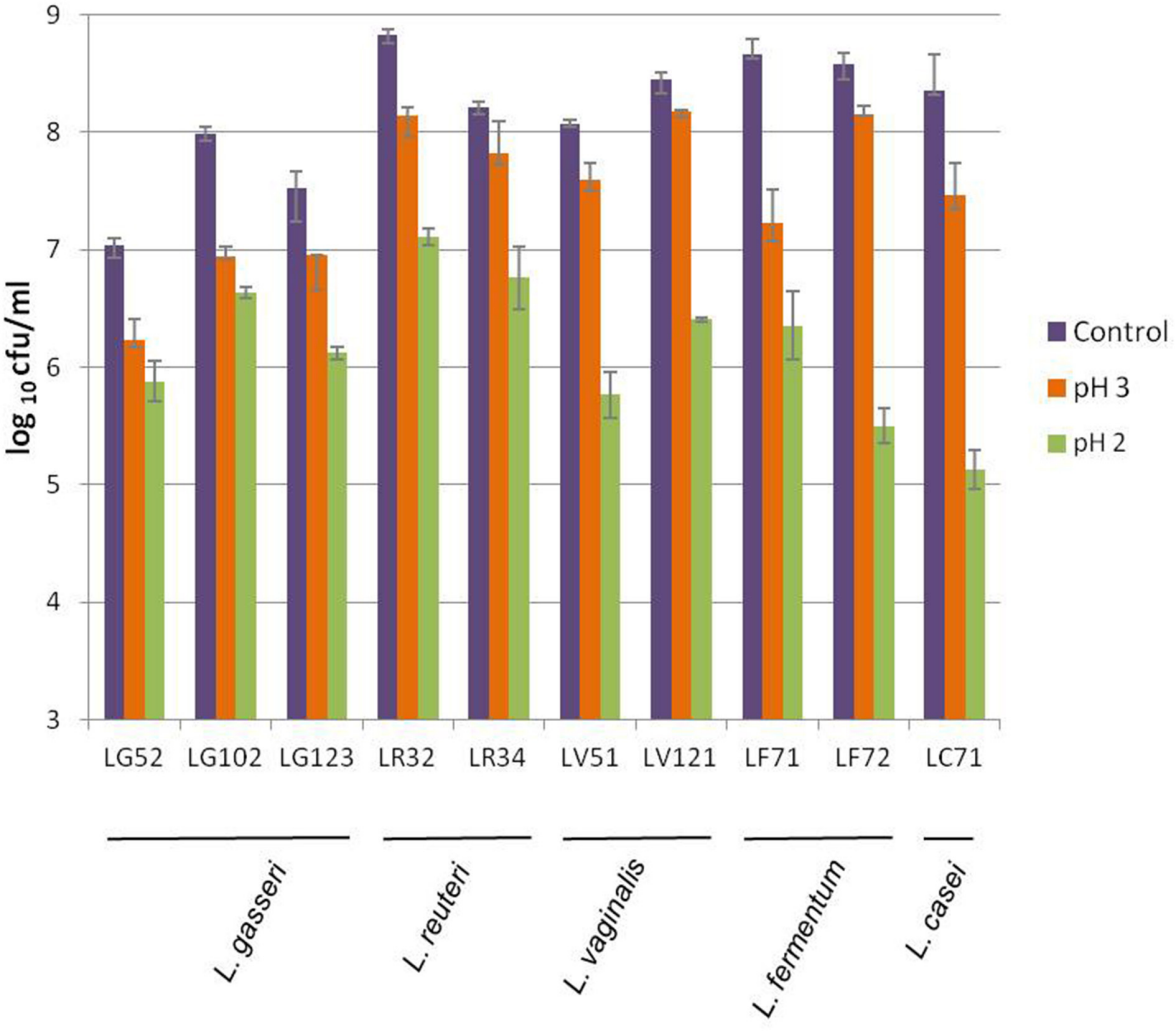
**Viability of the gastric *Lactobacillus* strains of this study to acidic conditions after exposure to pH 3 and pH 2 for 90 min, as compared to the control (pH 7.0)**.

As a whole, high tolerance to acidity was detected; similar plate counts were returned by control (pH 7) and treated samples after exposure to pH 4.5 and above (data not shown). At pH 3 reductions in counts varied among strains but in less than 1 logarithmic unit in all cases (Figure 1). However, at pH 2, reductions compared to controls counts were between 1 and 3 logarithmic units. The *L. gasseri* strains were among the most acid tolerant.

Resistance of the strains to bile varied widely, depending largely on the species. The *L. gasseri* strains were the most susceptible (MICs 0.25–1.5%), followed by those of *L. fermentum* (MIC = 1%). The most resistant strains were those of *L. reuteri*, *L. vaginalis* and *L. casei*, which grew in the presence of 4% bile.

**Figure 2:**
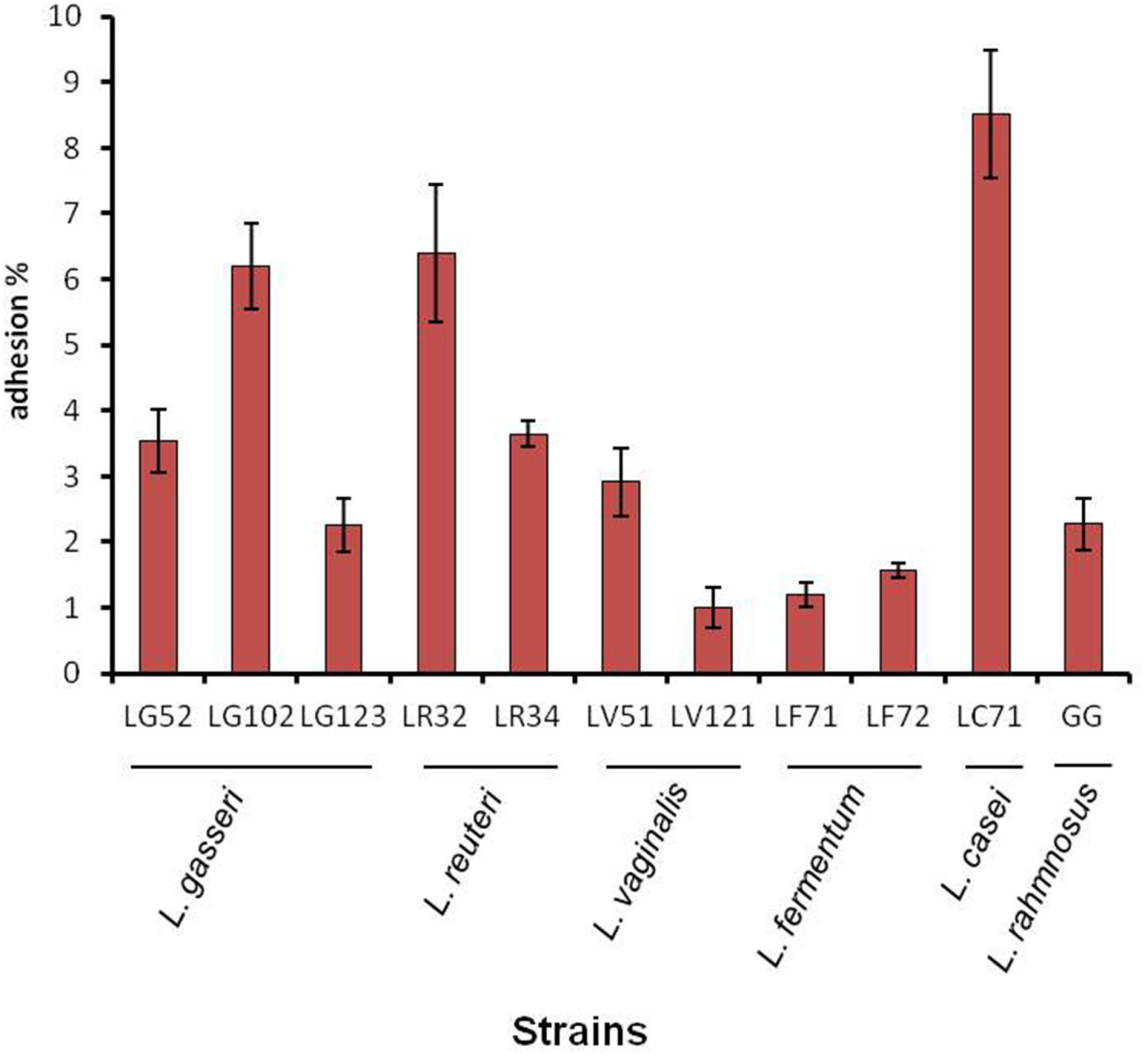
**Adhesion ability of gastric lactobacilli to the AGS human epithelial gastric cell line**.

The percentage of adhesion to gastric cells was strain-dependent but <10% for all strains - even for *L. rhamnosus* GG (Figure 2). The most adherent strain was *L. casei* LC71 (8.5%) followed by *L. reuteri* LR32 and *L. gasseri* LG102.

Regarding the bacteriocin-mediated antagonism assays, some gastric strains of the species *L. reuteri*, *L. gasseri* and *L. fermentum* were able to slightly inhibit the growth of the indicators in the agar spot test, although clear halos of inhibition using cell-free, neutralized supernatants were only observed for *L. gasseri* LG52 (Supplementary Table 1). The proteinaceous nature of the antimicrobial bacteriocin-like substance produced by this strain was confirmed after treatment of the cell-free supernatants with proteinase K and pronase, both of which eliminated the antibacterial effect.

**Figure 3:**
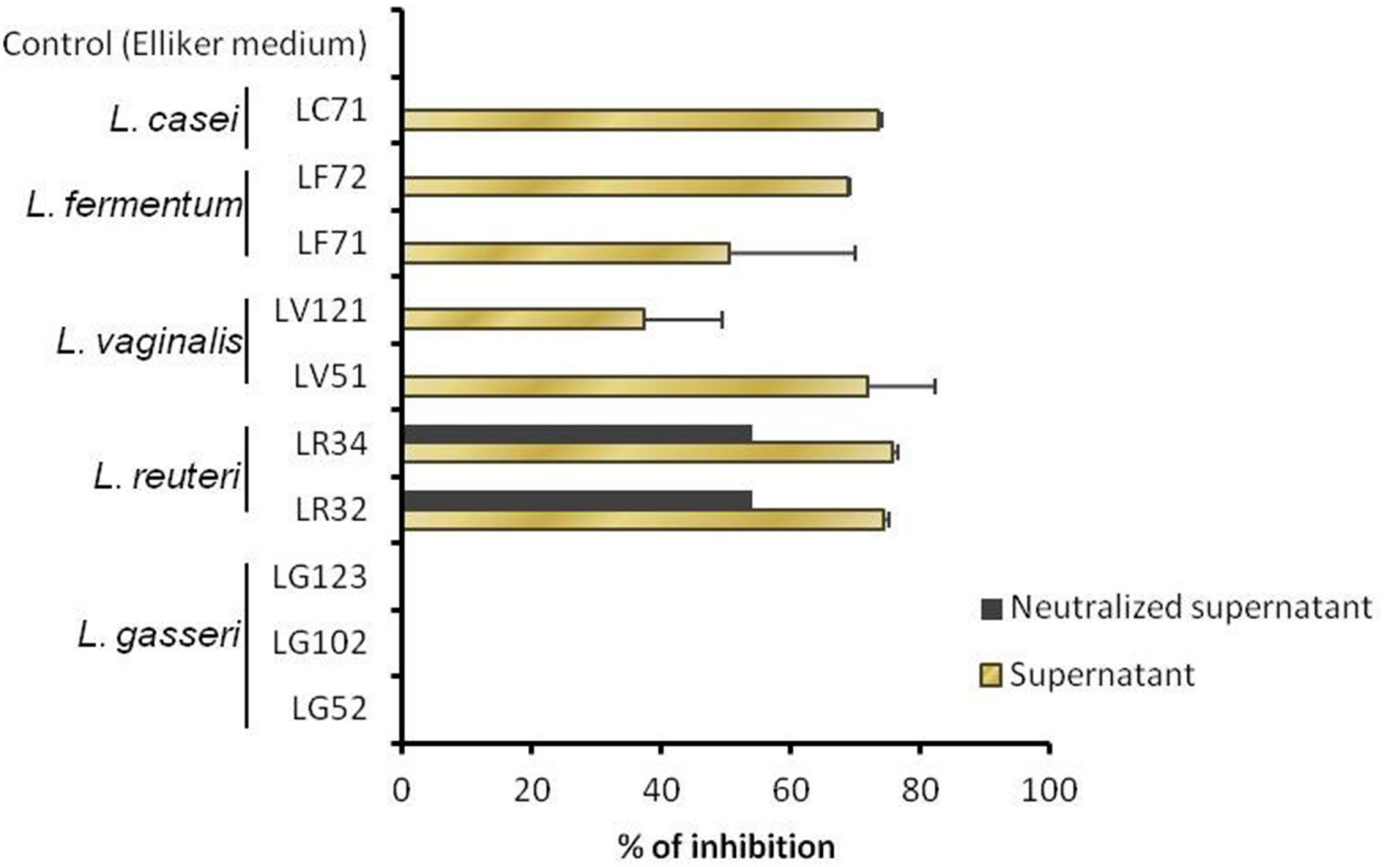
**Growth inhibition of *Helicobacter pylori*, as determined in liquid with non-adjusted and pH-neutralized supernatants of the *Lactobacillus* strains grown in Elliker medium**.

On the other hand, the *H. pylori* inhibitory test revealed all strains to have some antimicrobial activity against this pathogen, except for those of *L. gasseri* (Figure 3). The highest inhibitory values (~75%) were obtained for the two *L. reuteri* strains. When the supernatants were neutralized, *H. pylori* growth was suppressed only by the pH-adjusted supernatants pertaining to the *L. reuteri* strains, suggesting that the inhibition observed for the other strains was probably due to the production of organic acids. Further, the presence of genes associated with reuterin production (coding for the glycerol dehydratase unit) was revealed by PCR in the two *L. reuteri* strains.

As concerns H_2_O_2_ production, all *L. gasseri* strains showed slight production under aerobic conditions, while the *L. vaginalis* and *L. reuteri* strains clearly produced this substance under both aerobic and anaerobic conditions (Supplementary Table 1).

Strain-specific differences were also observed with respect to their TAA, as assessed by the LA test (Table 1). Comparable results were obtained using either whole cells or cell lysates. Remarkable activity (TAA >20%) was observed for *L. reuteri* LR32 and *L. vaginalis* LV51, and moderate activity (around a 15% reduction of lipid peroxidation) for *L. casei* LC71 and *L. reuteri* LR34. Neither the *L. fermentum* strains, nor *L. gasseri* LG123 and *L. vaginalis* LV121 showed any antioxidative activity under the experimental conditions of this assay.

**Table 1:** **Percentages of total antioxidative activity (TAA) determined by the linolenic acid test in intact cells and lysates of the gastric lactobacilli**.

**Species**	**Strain**	TAA^*^	
		**Whole cells (%)**	**Cell extracts (%)**
*L. gasseri*	LG52	5 ± 2^†^	3 ± 1
	LG102	16 ± 4	3 ± 1
	LG123	0	0
*L. reuteri*	LR32	22 ± 5	23 ± 7
	LR34	15 ± 3	14 ± 3
*L. vaginalis*	LV51	32 ± 8	21 ± 4
	LV121	0	0
*L. fermentum*	LF71	0	0
	LF72	0	0
*L. casei*	LC71	13 ± 4	15 ± 2

* *Following the definition by Hütt et al. (2006), the antioxidative effect was consider significant if the TAA value was >20%*.

† *Data are expressed as the mean value of three assays ± standard deviation*.

Atypical antibiotic resistance was not detected for any of the strains (Table 2). The MICs of the different antibiotics were always equal to or lower than the microbiological breakpoints defined by the Panel on Additives and Products or Substances used in Animal Feed (FEEDAP) of the European Food Safety Authority (EFSA, 2012). The exception was kanamycin, for which a MIC value of 64 μg/ml was observed for the two *L. fermentum* strains; the EFSA's breakpoint established for this species is one dilution lower.

**Table 2:** **Minimum inhibitory concentration (MIC) values of 16 antibiotics to the gastric lactobacilli strains**.

**Species**	**Strain**	Antibiotic^*^ (MIC as μg/ml)															
		**GEN**	**KAN**	**STP**	**NEO**	**TET**	**ERY**	**CLI**	**CHL**	**AMP**	**PEN**	**VAN**	**VIR**	**LIN**	**TRM**	**CIP**	**RIF**
*L. gasseri*	LG52	1	32	4	4	4	0.25	1	0.5	0.12	0.06	1	0.5	4	8	64	0.25
	LG102	4	32	4	16	2	0.12	0.5	4	0.5	0.12	1	0.5	2	4	64	1
	LG123	0.5	32	4	4	2	0.016	0.5	1	0.12	0.06	1	0.25	1	16	16	1
*L. reuteri*	LR32	1	64	16	4	4	0.12	0.03	4	1	0.12	>128	0.5	2	64	64	≤0.12
	LR34	0.5	16	8	0.5	8	0.5	0.12	4	2	0.5	>128	0.5	2	64	32	0.25
*L. vaginalis*	LV51	1	4	2	0.5	1	0.25	0.03	4	0.12	0.12	>128	0.12	1	0.25	32	≤0.12
	LV121	0.5	8	2	0.5	0.06	0.03	1	4	0.5	0.25	>128	0.12	2	0.25	64	0.25
*L. fermentum*	LF71	2	64	32	2	8	0.12	0.06	4	0.25	0.25	>128	0.25	2	1	8	1
	LF72	4	64	32	2	8	0.25	0.06	4	0.25	0.5	>128	0.5	2	16	16	0.5
*L. casei*	LP71	8	64	32	8	1	0.12	0.25	4	1	0.5	>128	1	2	0.5	4	1

* *GEN, gentamicin; KAN, kanamycin; STP, streptomycin; NEO, neomycin; TET, tetracycline; ERY, erythromycin; CLI, clindamycin; CHL, chloramphenicol; AMP, ampicillin; PEN, penicillin; VAN, vancomycin; VIR, virginiamycin; LIN, linezolid; TRM, trimethoprim; CIP, ciprofloxacin; RIF, rifampicin*.

### Technological traits

**Figure 4:**
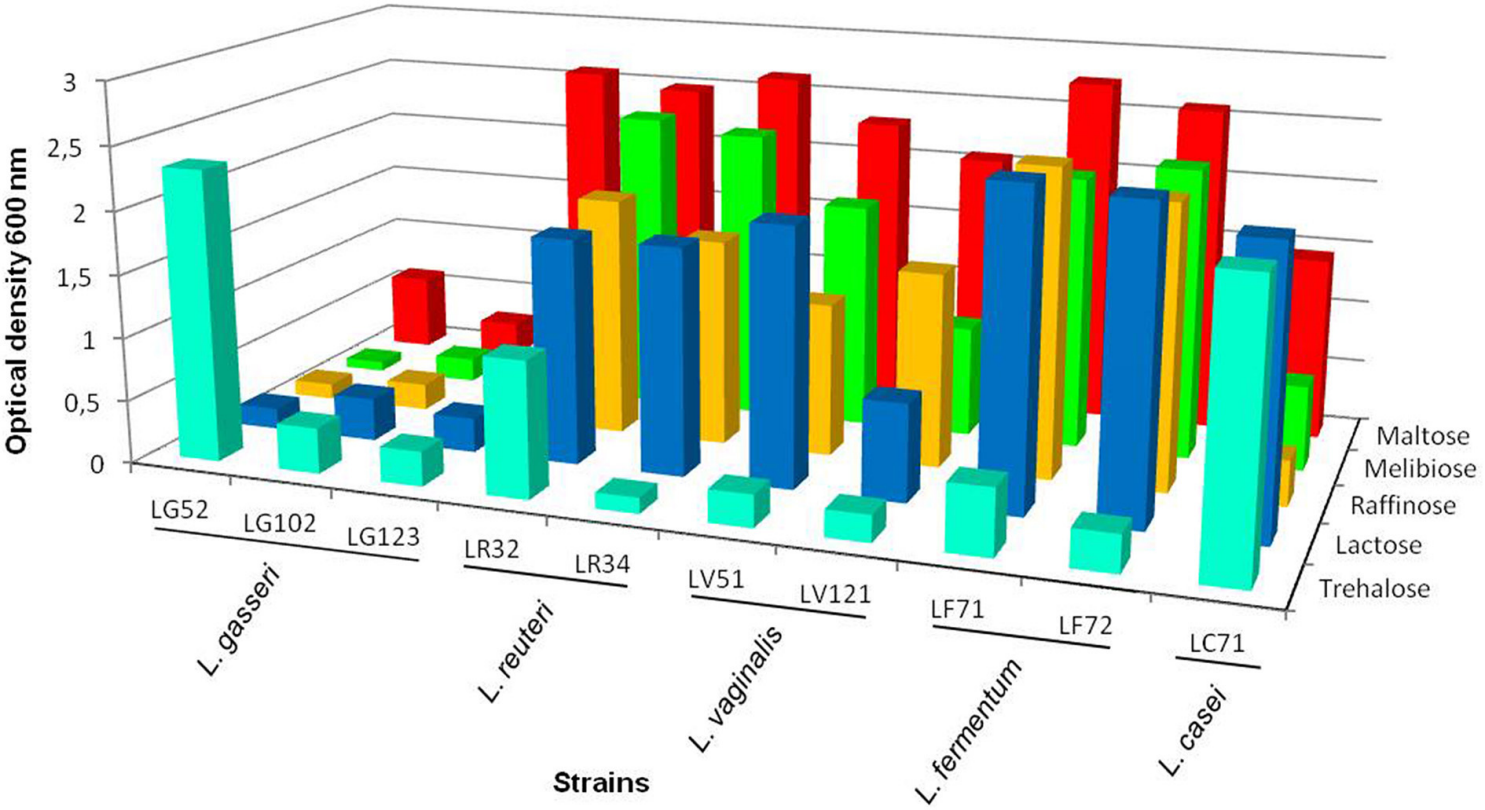
**Optical density at 600 nm of the lactobacilli strains grown in basal fermentation medium at 37°C for 48 h using different carbohydrates: maltose, melibiose, raffinose, lactose and trehalose as the carbon source (coefficient of variation <10%)**.

Differences were observed between the 10 strains in terms of their utilization of carbohydrates (Supplementary Table 2). All strains fermented maltose, and all but *L. gasseri* LG102 and LG123 fermented sucrose. Lactose was fermented by the single *L. casei*, the two *L. reuteri*, and the two *L. fermentum* strains. The growth capacity of the strains in basal fermentation medium with lactose, maltose, threhalose, melibiose and raffinose was further evaluated by recording the optical density after 24 and 48 h (Figure 4). In general, and in agreement with the previous results, the *L. gasseri* strains showed a reduced fermentation capacity compared to the others. Most of the gastric strains grew in the presence of maltose and lactose as the sole carbon source. The ability to grow in trehalose, however, was restricted to a few *Lactobacillus* strains (Figure 4). The *L. reuteri, L. vaginalis* and *L. fermentum* strains showed good growth rates in the presence of raffinose and melibiose.

Nineteen enzymatic activities were tested with the API-ZYM system. Moderate inter- and intra-species variability on the substrates utilized was observed (Supplementary Table 3). Some activities were shown by all or most strains and at high levels (Cys-, Val-, and Leu- arylamidase peptidases, and α- and β-galactohydrolase activities). In contrast, other activities (such as those of lipase, trypsin, α-quimotrypsin, α-mamnosidase, and α-fucosidase) were detected at low levels and only rarely. Glycosidic activities were also examined in cell-free extracts after growth of the strains in inducing carbohydrates. These activities may have a prominent role in key probiotic properties, such as utilization of prebiotics, colonization of the gastric epithelium, etc. The results are summarized in Table 3. With the exception of the *L. gasseri* strains (which did not grow in the presence of lactose) all showed high β-galactosidase activity. The *L. vaginalis* strains showed strong α-galactosidase activity, but so too did the *L. reuteri* and *L. fermentum* strains. The *L. casei* strain returned the highest α-glucosidase activity in presence of maltose.

**Table 3:** **Glycosidic activities of the *Lactobacillus* strains determined in cell-free extracts using *p*-nitrophenyl derivatives**.

**Species**	**Strain**	Activity (mU^*^/mg protein)				
		**α-glucosidase (maltose)**	**α-glucosidase (trehalose)**	**β-galactosidase (lactose)**	**α-galactosidase (melibiose)**	**α-galactosidase (raffinose)**
*L. gasseri*	LG52	–	36	–	–	–
	LG102	–	–	–	–	–
	LG123	<0.5	–	–	–	–
*L. reuteri*	LR32	<0.5	<0.5	865	63	8
	LR34	<0.5	–	574	6	24
*L. vaginalis*	LV51	12	–	551	1368	109
	LV121	<0.5	–	248	1170	106
*L. fermentum*	LF71	<0.5	–	145	795	12
	LF72	<0.5	–	318	2	96
*L. casei*	LC71	33	<0.5	86	–	–

* *The enzymatic Unit was defined as the amount of protein that releases 1 μmol of p-NP per min*.

*−OD 600 nm less than 0.7; activity was, therefore, not determined*.

Table 4 shows the strains' ability to grow in and acidify milk. After 24 h of incubation, the cell counts of all the strains increased slightly, but the figures for most (particularly *L. vaginalis* and *L. fermentum*) fell by 48 h. The exceptions were the *L. reuteri* strains, which reached values of around 2 × 10^8^ cfu/ml, and the *L. casei* strain, which showed the highest viable count (1 × 10^9^ cfu/ml) and the lowest pH at both sampling points. As expected, the titratable acidity followed an inverse trend with respect to pH, increasing as the pH decreased. A titratable acidity of around 81% of lactic acid equivalent after 48 h of incubation was recorded for *L. casei* LC71.

**Table 4:** **Growth and acidification of UHT milk by gastric lactobacilli strains**.

**Species**	**Strains**	Cell counts (cfu/ml)^*^		**pH^†^**		**Titratable acidity^‡^**	
		**24 h**	**48 h**	**24 h**	**48 h**	**24 h**	**48 h**
*L. reuteri*	LR32	4.1 × 10^7^	2.1 × 10^8^	6.02	5.75	21.5	24.0
	LR34	3.1 × 10^7^	2.4 × 10^8^	6.09	5.72	22.0	29.0
*L. vaginalis*	LV51	2.5 × 10^7^	1.6 × 10^7^	6.49	6.45	19.0	20.0
	LV121	1.5 × 10^7^	2.5 × 10^6^	6.50	6.49	19.0	21.0
*L. fermentum*	LF71	2.0 × 10^7^	9.9 × 10^6^	5.88	5.28	23.5	34.5
	LF72	3.7 × 10^7^	3.1 × 10^7^	5.95	5.53	23.5	33.0
*L. casei*	LC71	1.2 × 10^8^	1.3 × 10^9^	5.35	4.01	33.5	81.0

* *Inoculum ≈1 × 10^7^ cfu/ml*.

† *pH of the uninoculated milk 6.57*.

‡ *The titratable acidity is expressed as % lactic acid; uninoculated milk 18% lactic acid*.

## Discussion

The acidic environment of the gastric lumen limits the latter's microbial colonization to acidophilic and acid-resistant bacteria. Among these, different LAB species belonging mainly to the genera *Lactobacillus* and *Streptococcus* have been described (Adamson et al., 1999; Ross et al., 2005; Ryan et al., 2008a; Delgado et al., 2013). In this study, members of the genus *Lactobacillus* isolated from the stomach of healthy humans (see Delgado et al., 2013) were studied. Four out of the five *Lactobacillus* species examined (*L. gasseri*, *L. fermentum*, *L. vaginalis* and *L. reuteri*) have also been isolated from human gastric biopsies by other authors (Ryan et al., 2008a), which strongly suggests that they are common inhabitants of the gastric environment. Different strains belonging to the same species in a single individual were detected only occasionally. Based on the typing results, ten different strains were considered; these were characterized for their technological and probiotic properties. In general, they showed good tolerance and survival at low pH, indicating their capacity to survive in the human stomach. However, resistance to low pHs for longer periods of time (and colonization of the gastric epithelium) would be required to ensure persistence in the human stomach. Resistance to acidic conditions is a property of interest for the design and formulation of probiotic cultures. Such an ability would allow not only survival in the upper gastrointestinal tract, but also in fermented products (the most common vehicle for probiotics). The molecular mechanisms involved in such intrinsic resistance are currently unknown. The contribution of urease activity to this resistance however can be ruled out since all the present strains proved to be urease-negative (data not shown).

Since duodenogastric biliary reflux occasionally occurs, strains could also be resistant to bile. In fact, high resistance to bovine bile in lactobacilli strains from the gastric ecosystem has already been reported (Ryan et al., 2008a). Among the strains of this study, the *L. reuteri* proved to be the most resistant to Ox-gall. In contrast, as described for intestinal *L. gasseri* isolates (Delgado et al., 2007), the gastric strains of this species were rather susceptible to bile.

The strains were shown to adhere to gastric epithelial cells just as well, or even better than, the well-recognized adherent strain *L. rhamnosus* GG (Tuomola and Salminen, 1998). However, comparison of the results with those reported on the literature is difficult given the use of different cell lines (mostly colorectal Caco-2 and HT-29) and different cell-to-bacteria ratios employed.

Probiotics may be useful in the treatment of gastric dysbiosis, such as those caused by *H. pylori* infections. In fact, several studies have reported an inhibitory effect of probiotic lactobacilli on colonization and development of this pathogen (Johnson-Henry et al., 2004; Sykora et al., 2005; Francavilla et al., 2008). *H. pylori* colonizes the epithelium of the stomach and duodenum, occasionally invading the cells. Colonization seems to reduce systemic and cellular antioxidative defenses (Hütt et al., 2009). The antioxidative potential of the strains was therefore tested, a property that has already been reported for certain lactobacilli strains (Kullisaar et al., 2002). According to Hütt et al. (2006), total antioxidative (TAA) values >20% are considered noteworthy. In the present work, two strains, *L. reuteri* LR32 and *L. vaginalis* LV51, met this criterion. This protective property may be useful as a defense mechanism for the gastric mucosa, preserving the tissue from oxidant-induced damage.

The gastric lactobacilli were also screened for their antimicrobial activities against Gram-positive and Gram-negative bacteria. The production of bacteriocin-like inhibitory substances varies widely among LAB species and strains (de Vuyst and Leroy, 2007). After successive solid and liquid medium assays, a single *L. gasseri* strain (LG52) was consistently shown to be bacteriocin producer. However, whether LG52 produces an active bacteriocin under gastric conditions remains yet to be investigated. Another potential source of inhibitory effects is H_2_O_2_, which in certain environments might be more important than the production of organic acids. The stomach is a microaerobic environment and the production of H_2_O_2_ might be greater under such conditions than in anaerobiosis. This prompted us to evaluate the production of H_2_O_2_ under aerobic and anaerobic conditions. The two *L. reuteri* strains and *L. vaginalis* LV51 were shown to produce this compound under both conditions. These strains must have H_2_O_2_ detoxification mechanisms that allow them to protect themselves from its toxic effects. Superoxide dismutases, peroxidases and dehydrogenases are all able to degrade H_2_O_2_, and have been described in lactobacilli species (Kullisaar et al., 2002; Hütt et al., 2006; Martín and Suárez, 2010). These enzymes contribute toward the antioxidative cell defense system. In the present work, the strains with the largest H_2_O_2_ production capacity were those with the greatest antioxidative activity.

The inhibition of *H. pylori* by the two *L. reuteri* strains was probably due to the production of reuterin; certainly, a gene essential for its production was detected. Reuterin is a potent antimicrobial agent that inhibits both Gram-positive and Gram-negative bacteria. *L. reuteri* also produces other potent antimicrobial compounds, such as reutericin 6 and reutericyclin, but these have no effects on Gram-negative bacteria (Gänzle, 2004). The inhibition of *H. pylori* by lactobacilli has already been reported (Sgouras et al., 2004; Hütt et al., 2006; López-Brea et al., 2008; Ryan et al., 2008b). However, most authors attribute the observed inhibitory effects to live metabolizing cells. In contrast, the present work provides evidence of the inhibitory effect of *L. reuteri* culture supernatants against *H. pylori*. Wherever this inhibitory activity comes from, the potential use of these human stomach-derived *L. reuteri* strains as probiotics for protecting against *H. pylori* infection should be considered.

While *L. gasseri* seems to be more prevalent in the gastric ecosystem than *L. reuteri*, the strains of the latter species displayed more probiotic-relevant properties and/or higher activity levels. The antimicrobial—and especially anti-*H. pylori*—activity of the *L. reuteri* strains, together with their antioxidative effects, might allow them to protect the gastric mucosa from infection and damage. The *L. reuteri* strains also showed some technological traits that would allow their inclusion in fermented dairy products. Strains of other species further showed desirable traits to be recommended as probiotic candidates. As an example, *L. vaginalis* LV51 showed the strongest α-galactosidase activity. This would be highly desirable in soy-derived products to hydrolyse the α-galactosides (mainly raffinose and stachyose) capable of causing gastrointestinal discomfort and flatulence (LeBlanc et al., 2008).

## Conclusions

In summary, this work reports a genotypic, technological and probiotic description and characterization of a group of lactobacilli from the human stomach. *In vitro*, some of the gastric strains (particularly the *L. reuteri* strains) showed a vast array of desirable properties to be considered as promising probiotic candidates. Additionally, they showed appropriate technological traits to be included in dairy or other fermented functional foods as adjunct cultures. The efficacy of such probiotics for the treatment and/or prevention of gastric microbial dysbiosis should be carefully evaluated *in vivo* through controlled clinical trials.

## Author contributions

Susana Delgado and Baltasar Mayo contributed with the conception and design of the study. Susana Delgado and Analy M. O. Leite were involved in the experimental determinations. Patricia Ruas-Madiedo was in charge of the adhesion experiments and in the maintenance of the gastric cell line. Susana Delgado and Baltasar Mayo interpreted the data and drafted the manuscript. Patricia Ruas-Madiedo performed a critical revision of the manuscript. All authors approved the final version of the article.

### Conflict of interest statement

The authors declare that the research was conducted in the absence of any commercial or financial relationships that could be construed as a potential conflict of interest.
