# Supplementary material for: Probiotic and technological properties of Lactobacillus spp. strains from the human stomach in the search for potential candidates against gastric microbial dysbiosis
Source: Front Microbiol. 2015 Jan 14;5:766. doi: 10.3389/fmicb.2014.00766 (PMC4294198; doi:10.3389/fmicb.2014.00766)
Supplement: Supplementary file 1 [file Table1.PDF]

Supplementary Table 1. Production of bacteriocin-like substances, and H_2O_2 under aerobic and anaerobic conditions by gastric lactobacilli.

**Species**	**Strain**	**Bacteriocins**				**H_2O_2**	
		Lactobacillus sakei CECT 906		*Lactococcus lactis* IL 1403			
		OC	CFS	OC	CFS	Aerobic	Anaerobic
L. gasseri	LG52	+	+	-	-	+/-	+
	LG102	-	-	-	-	+/-	-
	LG123	-	-	-	-	+/-	-
L. reuteri	LR32	-	-	+/-	-	+	+
	LR34	-	-	+/-	-	+	+
L. vaginalis	LV51	-	-	-	-	+	+
	LV121	-	-	-	-	+	+/-
L. fermentum	LF71	+/-	-	-	-	-	-
	LF72	+	-	-	-	-	-
L. casei	LC71	-	-	-	-	-	-

+/-, slight production/ inhibition

OC, Overnight culture (agar spot test)

CFS, Cell free and neutralized supernatant (well-diffusion assay)
