# Supplementary material for: Probiotic and technological properties of Lactobacillus spp. strains from the human stomach in the search for potential candidates against gastric microbial dysbiosis
Source: Front Microbiol. 2015 Jan 14;5:766. doi: 10.3389/fmicb.2014.00766 (PMC4294198; doi:10.3389/fmicb.2014.00766)
Supplement: Supplementary file 2 [file Table2.PDF]

Supplementary Table 2. Carbohydrate fermentation profiles of the *Lactobacillus* strains isolated from the human stomach.

Species	Strains	ARA	XIL	GAL	MAL	CEL	TRE	PAL	SAC	LACT	MEL	MAN	MELE	INO	MANI	ARB	SOR	TAG	AMI	GLU	SAL
L. gasseri	LG52	-	-	-	+	+	+	-	+	-	-	-	-	-	+	+	-	-	+	-	+
	LG102	-	-	-	+	-	-	-	-	-	-	-	-	-	-	-	-	-	-	-	-
	LG123	-	-	-	+	-	-	-	-	-	-	-	-	-	-	-	-	-	-	-	-
L. reuteri	LR32	+	-	+	+	-	-	-	+	+	+	-	-	+	-	-	-	-	-	+	-
	LR34	+	-	+	+	-	-	-	+	+	+	-	-	-	-	-	-	+	-	-	-
L. vaginalis	LV51	-	-	-	+	-	-	-	+	-	-	-	-	-	-	-	-	-	+	-	-
	LV121	-	-	-	+	-	-	-	+	-	-	-	-	-	-	-	-	-	-	-	-
L. fermentum	LF71	-	-	+	+	+	+	-	+	+	-	+	-	-	-	+	-	+	+	-	+
	LF72	-	-	+	+	-	+	-	+	+	+	+	-	+	-	-	-	+	-	-	-
L. casei	LC71	-	-	+	+	+	+	-	+	+	-	+	+	-	+	+	+	+	+	+	+

ARA: arabinose; XIL: xylose; GAL: galactose; MAL: maltose; CEL: cellobiose; TRE: trehalose; PAL: palatinose; SAC: sucrose; LACT: lactose; MEL: melibiose; MAN: mannose; MELE: melezitose; INO: inositol; MANI: mannitol; ARB: arbutin; SOR: sorbitol; TAG: tagatose; AMI; amygdalin; GLU: gluconate; SAL: salicin.

None of the strains fermented any other sugar and polyalcohol of the API-50 CH strip.
