# Supplementary material for: Probiotic and technological properties of Lactobacillus spp. strains from the human stomach in the search for potential candidates against gastric microbial dysbiosis
Source: Front Microbiol. 2015 Jan 14;5:766. doi: 10.3389/fmicb.2014.00766 (PMC4294198; doi:10.3389/fmicb.2014.00766)
Supplement: Supplementary file 3 [file Table3.PDF]

Supplementary Table 3. Enzymatic activities of the gastric lactobacilli as scored with the API-ZYM system.

Species	Strain	Enzymatic activity*																		
		Alkaline phosphatase	Esterase	Esterase lipase	Lipase	Leucine arylamidase	Valine arylamidase	Cystine arylamidase	Trypsin	α-chymotrypsin	Acid phosphatase	Naphthol-AS-BI-phosphohydrolase	α-galatosidase	β-galactosidase	β-glucuronidase	α-glucosidase	β-glucosidase	N-acethyl-β-glucosamidase	α-mamnosidase	α-fucosidase
	LG52	-	2.5	-	2.5	40	40	10	-	-	10	30	2.5	2.5	40	10	30	10	2.5	2.5
L. gasseri	LG102	-	2.5	-	-	40	0	40	2.5	-	2.5	40	-	-	2.5	-	2.5	-	-	-
	LG123	2.5	40	-	-	40	40	40	-	-	2.5	20	20	-	-	-	20	30	-	-
L. reuteri	LR32	2.5	40	2.5	-	40	40	10	-	-	30	10	40	40	-	40	-	-	-	-
	LR34	-	-	-	-	-	-	-	-	-	2.5	2.5	10	30	-	2.5	-	-	-	-
L. vaginalis	LV51	2.5	10	20	2.5	40	40	10	-	-	20	10	40	40	2.5	-	-	-	-	-
	LV121	-	20	10	-	40	20	20	-	-	20	30	40	40	-	-	-	-	-	-
L. fermentum	LF71	-	10	-	-	40	20	40	-	-	10	2.5	40	40	-	-	2.5	2.5	2.5	2.5
	LF72	-	10	30	-	30	40	10	-	-	20	0	40	40	40	10	-	-	-	-
L. casei	LC71	40	10	10	2.5	40	40	30	-	40	10	10	-	20	-	40	2.5	-	-	-

*Units of activity are expressed as nmol of substrate hydrolyzed after a 4-h incubation period at 37ºC.

-: Activity was not detected.
