# Supplementary figures and images for: Probiotic and technological properties of Lactobacillus spp. strains from the human stomach in the search for potential candidates against gastric microbial dysbiosis
Source: Front Microbiol. 2015 Jan 14;5:766. doi: 10.3389/fmicb.2014.00766 (PMC4294198; doi:10.3389/fmicb.2014.00766)

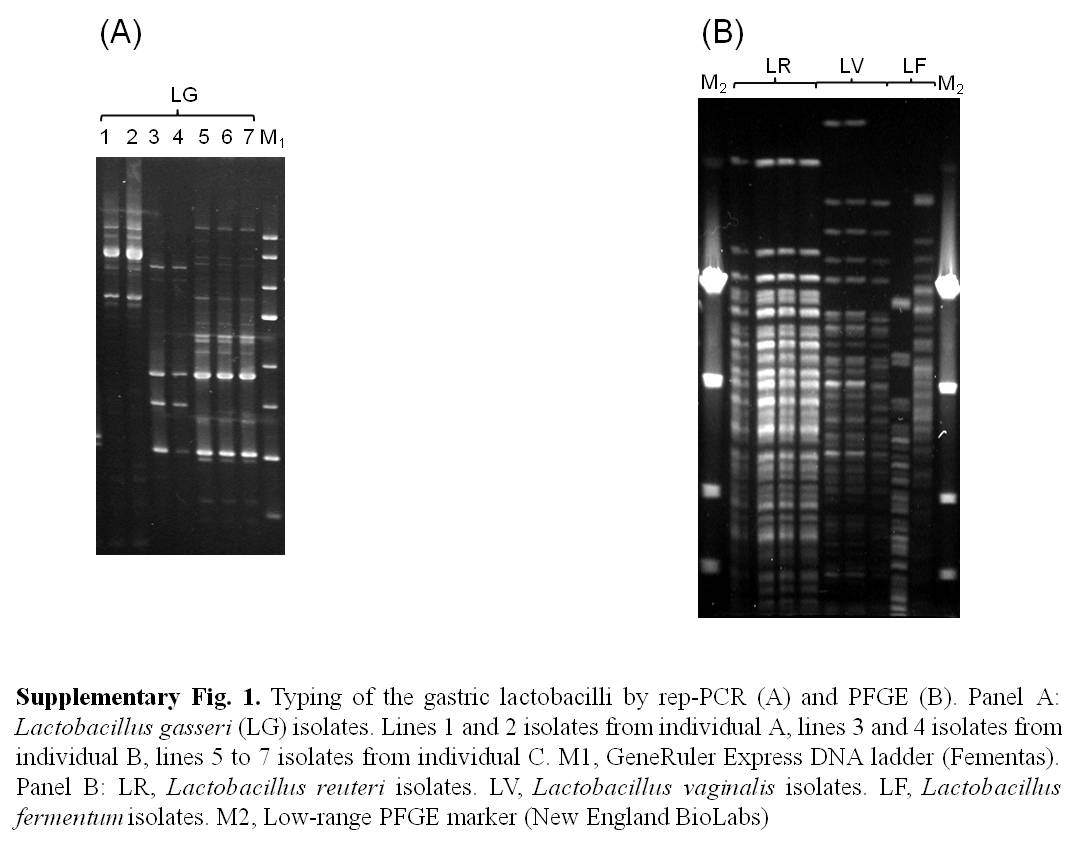

Supplement: Supplementary file 4 [file Image1.JPEG]
